# Developing a Q Set Using the Modified Delphi Technique to Investigate ICU Nurses’ Perspectives on Working with Non-ICU Nurses

**DOI:** 10.3390/healthcare13090973

**Published:** 2025-04-23

**Authors:** Dalia Sunari, Adel Bashatah

**Affiliations:** Department of Nursing Administration and Education, College of Nursing, King Saud University, Riyadh 11421, Saudi Arabia

**Keywords:** Delphi technique, Q methodology, Q set, ICU nurses, non-ICU nurses

## Abstract

**Background/Objectives**: ICU nurses manage complex clinical situations of critically ill patients, including rapid patient deterioration and multiple invasive lines. The complexity intensifies in catastrophes when non-ICU nurses are trained on short notice and deployed to support ICU nurses. This article details the rigorous development of the Q set, which is essential for ensuring methodological robustness and validity in a Q methodology study exploring ICU nurses’ perspectives on working with non-ICU nurses. **Methods**: A modified Delphi approach was adopted for the expert consensus on the selection of statements, which ensured an unbiased Q sample construction. The methodology involved (1) concourse generation based on the literature review and semi-structured interviews of ICU nurses and (2) Q sample refinement via expert consensus. **Results**: The process extracted 152 statements in two Delphi rounds with ICU experts (head nurses, bedside nurses, and nursing faculty). The first round finalized 13 and excluded 8 statements. The second round further added 27 and excluded 5 statements. Thus, the final Q sample comprised 40 statements, which were reviewed by a Q methodology expert. **Conclusions**: The study improved the rigor, precision, and transparency of Q sample construction in ICU nursing research. The incorporation of the expert consensus minimized the bias to accurately examine ICU nurses’ perspectives. The results offer valuable insights into non-ICU nurses’ integration in critical care settings to guide staffing policies, training, and inter-professional collaborations.

## 1. Introduction

Rapidly changing conditions of critically ill patients demand efficient functioning in stressful environments. It requires high proficiency of intensive care unit (ICU) nurses in the simultaneous management of multiple invasive lines (endotracheal tubes, central venous pressure lines, and arterial lines) [1]. The complexity intensifies when ICU nurses collaborate with other healthcare specialties during disastrous emergencies requiring urgent ICU staffing for ventilated patients. In this situation, non-critical care unit nurses are trained on short notice to serve as ICU nurses [2,3]. The swift transition is often positively viewed by non-ICU nurses. However, it poses a psychological impact as their effective working requires substantial support from existing ICU staff [4]. It could result in increased burnout levels of ICU nurses as they provide support to non-ICU nurses in addition to managing critically ill patients. Therefore, to avoid medical errors and improve care quality, comparatively stable and less complicated cases are assigned to non-critical care nurses [4,5,6].

Multiple studies have investigated non-critical care nurses’ experiences in ICUs during crises [4,7,8]. However, the literature lacks information about its impacts on ICU nurses. Particularly, the perception of ICU nurses regarding collaborative working with non-ICU nurses for attending to critically ill patients requires further elaboration. The main research question of the Q methodology study is the following: how do critical care nurses perceive working with non-critical care nurses in critical care settings? In order to answer this question, a Q set needed to be developed and validated. Therefore, a modified Delphi method was adopted to facilitate expert consensus during statement selection to ensure the construction of a robust and unbiased Q sample. Q-sample statements are traditionally selected by the researchers [9,10]. However, the modified Delphi technique in conjunction with the Q methodology could generate concourses and pilot Q samples without researcher bias [11,12]. Thus, this modified approach was followed to ensure transparency and detailed construction of Q samples for the current Q methodology study. The aim of this study is to construct a comprehensive Q-set and establish its content validity through the application of the Delphi technique. This paper will discuss the development of the concourse and the initial Q set that was used for the Delphi technique. It also details the rounds of the Delphi technique.

## 2. Materials and Methods

### 2.1. Construction of the Q Sample

Q sample construction mainly involved the generation of a concourse using a modified Delphi technique. Initially, the identification of a range of opinion statements was carried out through comprehensive literature screening and semi-structured interviews of ICU nurses who had worked with non-ICU nurses in ICU settings. It helped in devising a concourse for a diverse population. The second step involved the application of the modified Delphi technique for an expert consensus on finalizing the statements.

### 2.2. Concourse Generation

**Figure 1:**
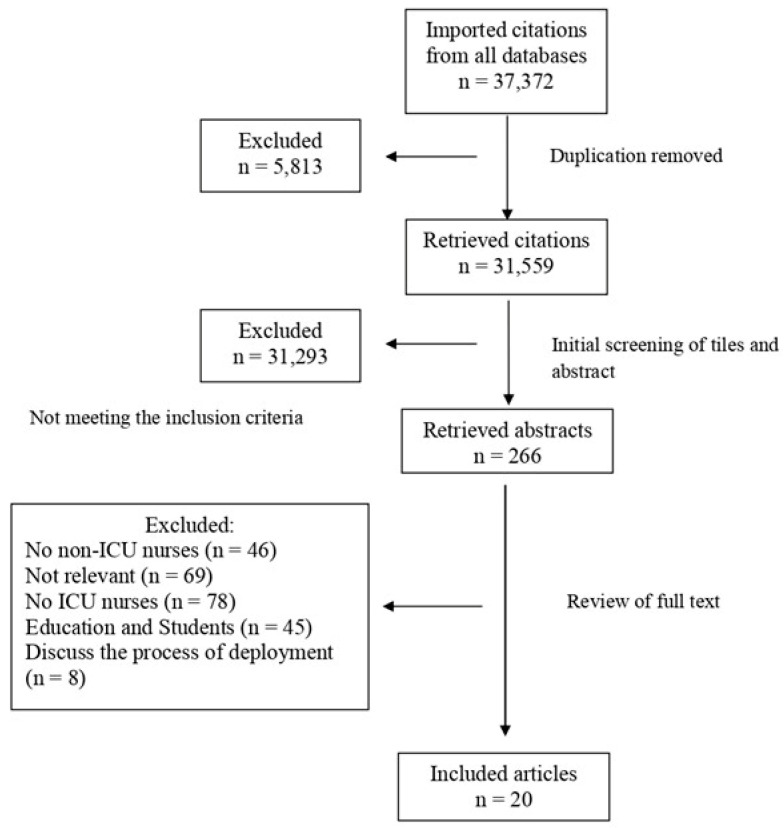
Summary of findings from the search of the literature. Source: Elaborated by the authors.

The concourse was generated by reviewing the literature on various databases [EBSCO (CINAHL Complete, MEDLINE, Sociology Source Ultimate, ERIC) and Web of Science (SCIELO, MEDLINE, WOS, PQDT, ARCI, PPRN, CCC, KJD, and DIIDW)]. The literature screening was carried out on 30 October 2023, which yielded 31,559 articles. Only 20 of the screened articles indirectly discussed ICU nurses’ perspectives on working with non-ICU colleagues (Figure 1). The review was conducted using the Rayaan application. Studies were included if they met the following criteria: (1) utilized any research design, whether qualitative or quantitative; (2) involved critical care nurses working in either adult or pediatric intensive care units; (3) focused on interactions, experiences, perceptions, or behaviors between nurses; or (4) were conducted within any type of ICU setting. Studies were excluded if they focused on interactions between ICU and non-ICU nurses that involved nursing interns or students, as the scope of this review was limited to registered nurses with established clinical roles. A total of 50 statements were extracted from the literature.

Given the limited number of relevant articles, additional sources of data were needed. Therefore, semi-structured interviews with ICU professionals were conducted and additional statements were gathered. Purposive sampling was employed to recruit ten ICU professionals from multiple hospitals, including eight bedside ICU nurses and two ICU nurse educators. This sampling strategy ensured maximum variation and comprehensive coverage of experiences and perceptions related to the integration of non-ICU nurses within ICU settings. Interviews were conducted via Zoom, audio-recorded with verbal consent, and transcribed verbatim. Participants were encouraged to share their experiences, feelings, and perceptions openly. Data collection continued until saturation was achieved, indicated by no emergence of new perspectives and ideas.

From the interviews, an additional 102 statements were generated, increasing the total concourse to 152 statements. All interview transcripts were analyzed by a single researcher using initial open coding, systematically identified recurring patterns, viewpoints, and overarching themes representing participants’ lived experiences and perceptions.

As part of the open coding process, the authors became familiar with the interview transcripts. Opinion statements were highlighted and then extracted. For instance, Participant 9 said, “I had to take on some teaching responsibilities. I must say, the ward staff were under a lot of mental stress—they were really afraid of dealing with ICU patients”. The statements derived were “ICU nurses are responsible for sharing their knowledge and experience with inexperienced nurses” and “I empathize deeply with the non-ICU nurses working in such a complex environment”. Another participant (3) stated, “We assign them a patient and then explain our ICU routines, including doctors’ rounds. We also go over what to do if a surgical or trauma patient arrives—we need to explain everything to them”. From this, the extracted statement was “Non-ICU nurses require plenty of preparation before working with critically ill patients”. A representative sample of the coded data and extracted statements is provided in Supplementary Materials.

Following the open coding, statements were systematically extracted and organized. Each statement was critically reviewed for redundancy, overlap, and ambiguity. Duplicated or overlapping statements were merged or eliminated, and unclear statements were refined for clarity, simplicity, and alignment with a single core idea, resulting in a distinct and representative final Q-set.

**Figure 2:**
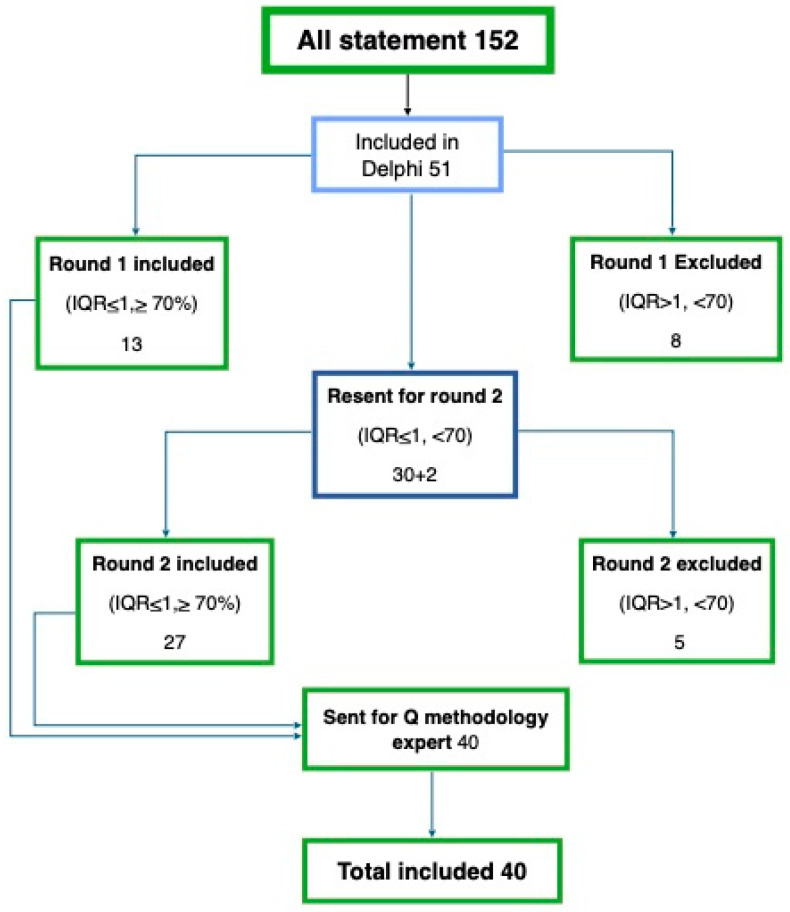
Statement development process. Source: Elaborated by the authors.

To ensure research credibility and trustworthiness, methodological rigor was maintained through data triangulation, member-checking, and a detailed audit trail documenting the analytic process. Informed consent was secured from all participants, and confidentiality was rigorously maintained, underscoring the ethical integrity and transparency of the research process. Finally, 51 statements were finalized and included in the modified Delphi technique (Figure 2).

### 2.3. Modified Delphi Technique

The modified Delphi technique was retrieved from the traditional Delphi method to improve flexibility and efficiency while maintaining the core principles of expert consensus-building through structured feedback. The traditional Delphi technique typically begins with open-ended questions, whereas the modified version includes predefined questions/statements. These questions/statements focusing on specific objectives are often derived from the literature for a smooth process [13]. The modified approach reduces the number of required rounds for a targeted and time-efficient process. The modified Delphi technique has wide applications in various disciplines such as public policy, healthcare, business, and education. Participants’ anonymity is the key aspect of the Delphi method, which alleviates the influence of dominant individuals for unbiased contributions [14]. Anonymity mitigates the likelihood of groupthink and influential personalities’ impacts to encourage experts’ objective consensus. The methodology involves multiple feedback rounds where experts are provided with a response summary of previous rounds. It facilitates group insights-based reassessment and refinement of their opinions [11]. This repetitive technique yields a more accurate consensus on collective judgment. Thus, the Delphi technique helps in achieving consensus among experts, which makes it a valuable tool in situations with limited empirical data availability requiring essential expert judgment [11,13,15]. During this study, a two-round technique was employed to further reduce the number of statements. It assisted in reaching expert consensus regarding the inclusion of statements in the Q sample. The modified Delphi process was carried out from July to September 2024. The IRB committee of King Saud University, Riyadh, KSA, provided the ethical approval.

### 2.4. Panel Selection

The modified Delphi technique relies on an expert panel that reaches a consensus through multiple rounds of questionnaires [13,16]. The following expert panel inclusion criteria were adopted during the study: (1) ICU nurses with experience working with non-ICU nurses in an ICU setting, (2) ICU experience (minimum of two years) to ensure familiarity with ICU norms and procedures, and (3) ICU experts such as ICU head nurses or ICU faculty members. The constituted panel included experienced members with ICU and research expertise. The expert panel comprised bedside ICU nurses, ICU head nurses, and critical care faculty members. All the members had exposure to ICU placements and non-ICU nurses in the ICU setting. Moreover, their direct ICU experience, mentorship, and integration with non-ICU nurses provided a comprehensive perspective regarding the research question. The final panel consisted of 21 experts. Their anonymity was maintained to mitigate dominant personalities’ influence during decision-making. Experts were contacted and invited via WhatsApp or phone call. Upon agreeing, a survey link was sent through WhatsApp or email based on their preference.

### 2.5. Modified Delphi Rounds

#### 2.5.1. Round One

During the first round, 51 statements were sent to the participants via Google Forms, whereas the link was provided through a WhatsApp message or an invitation email. Experts were asked to rate each statement on a 3-point Likert scale based on its relevance to ICU nurses working with non-ICU nurses in ICU settings (1 = not relevant to the study, 3 = relevant to the study). Moreover, participants were invited to comment on unclear statements and propose new ones if they felt there were missing important aspects. SPSS version 29 was employed to analyze the responses. Descriptive statistics of each statement, including the median, percentage agreement, and interquartile range (IQR), were computed. Statements with less than one IQR were considered to reach a consensus with the agreement of 70% of participants on the relevance of the statement [16]. Statements meeting the required criteria were directly included in the Q sample, whereas others were modified according to experts’ feedback and included in the next round. Furthermore, new statements suggested by panelists were reviewed in subsequent rounds and included if necessary.

#### 2.5.2. Subsequent Rounds

The statements failing to achieve consensus (IQR < 1 and less than 70% agreement) were revised and resent to the panel for further evaluation in subsequent rounds. Panelists suggested new statements in the first round that were also added in subsequent rounds. The process was repeated until a consensus was reached on the remaining statements. It ensured an accurate reflection of expert opinions in the final Q sample.

## 3. Results

### 3.1. Generation of the Concourse

The concourse was formed by extracting 152 statements from the interviews. The interviews (20 to 40 min each) were recorded and transcribed for the extraction of opinion statements. Then, similar statements were deleted and the clarity of the remaining statements was reviewed. Statements with multiple/complex ideas were simplified to have only one idea/feeling in each statement. For example, the statement “It is hard to work next to non-nurse as it makes me stressed and worried but if they are interested in ICU jobs it is much easier” was divided into two statements such as “ICU nurses feel stressed when they are working with non-ICU nurses” and “It is easy to deal with non-ICU nurses who are interested in ICU units”. The duplicates were removed, and each statement had a negative or positive format to select the text with better clarity and best wording. Finally, 51 statements were included in the study, and similar statements were grouped for the analysis. The groups were named “Preparation and Training”, “Collaborations and Communication”, “Workload, Roles, and Dynamics”, “Feelings and Emotions”, “Patient Safety and Care Quality”, and “Opportunities and Trust” (Table 1).

### 3.2. Q Sample Preparation with the Modified Delphi Technique

The round one online survey was completed by 21 experts, which included ICU head nurses or nurse managers (28.6%, n = 6), ICU bedside nurses (33.3%, n = 7), and nursing faculties (38.1%, n = 8) specialized in critical care nursing. The majority of participants were female (95%, n = 20), while all participants had over five years of professional experience in their respective fields. Most of the participants held a bachelor’s degree in nursing (57.1%, n = 12), while six participants held a PhD (28.6%, n = 6), and two held a master’s degree in nursing (14.2%, n = 3).

Researchers recruited 16 experts through direct invitation, whereas five experts were recruited via participant referral. The experts were contacted by phone to explain the Delphi process and obtain their agreement. The Delphi process was completed in around 3 months. Round one was initiated in July 2024 and participants were asked to complete the online link in two weeks. Round two started in September 2024 and participants were allowed to complete the online link in three weeks. However, the panel retention rate remained at 57.14% for round two, where only 12 experts completed the survey. Non-responders of Round Two included five bedside nurses and four head nurses. Two Emails and WhatsApp messages were sent to these participants as reminders, but they declined to continue their participation due to busyness.

### 3.3. Round One

Overall, 13 statements reaching consensus were included in the Q sample (IQR ≤ 1, reaching over 70% relevance), whereas 8 statements were excluded (IQR >1, lower than 70% relevance). The statements that were included did not receive major comments. Participants only requested minor wording modifications, such as replacing “non-ICU nurses” with “non-ICU specialized nurses”, which could have elongated the statements. Moreover, participants were very familiar with the fact that “non-ICU nurses” refer to nurses from other departments (medical, outpatient, and surgical clinics). Therefore, these suggested amendments were not adopted. The remaining 30 and 2 additional statements were subjected to the second round. However, experts found one statement regarding “sarcastic behavior to patient care providers” to be offensive to the nursing profession. However, it was included for round two as it held a good point of presenting the opinion of ICU nurses. The additional statements covered issues of working with non-ICU nurses, including “(1) Working with sufficiently trained non-ICU nurses decreases ICU nurse struggle, and (2) Years of experience of non-ICU nurses make no difference when floating to ICU units”.

### 3.4. Round Two

In total, 27 statements reaching consensus were included in the Q sample (IQR ≤ 1, reaching over 70% relevance). Three statements were excluded for lower relevance (IQR >1, lower than 70% relevance), whereas two statements were excluded based on expert recommendations. Round two comments mainly focused on the repetition of ideas and meanings. Finally, clear and simple statements were included in the Q set sample. Panelists recommended changing the term “ethical issues” to “medical errors” in one statement. The included statements (40) were sent to the Q methodology expert for final review and modifications. The expert suggested alterations in opposing statements, such as “ICU nurses consider non-ICU nurses as a beginner in ICUs” and “ICU nurses consider non-ICU nurses as an assistant to them in ICUs”. However, some suggestions were not related to the main research ideas, such as “ICU nurses are able to predict patient outcomes”. These statements mainly focused on ICU nurses’ nursing care rather than non-ICU nurses. Therefore, such opposing statements about ICU nurses’ nursing care were not included in the study. A Q methodology expert might have suggested these modifications as he/she was not related to the nursing or medical fields. However, the expert’s opinion facilitated the statement writing (Table 1).

The finalized statements (40) were categorized into multiple themes according to ideas and opinions. The theme “opportunities and trust” had the highest frequency with 8 statements out of 40. Three themes, “Patient Safety and Care Quality”, “Workload, Roles, and Dynamics”, and “Preparation and Training”, contained seven statements. The remaining two themes, “Collaborations and Communication” and “Feelings and Emotions”, contained six and five statements, respectively.

## 4. Discussion

The development of an unbiased and comprehensive Q sample is mandatory to obtain accurate perspectives of ICU nurses working with non-ICU nurses in critical care settings. During this study, a structured approach was adopted to construct a Q sample by incorporating insights from the literature and first-hand experience gathered via semi-structured interviews. A modified Delphi technique was employed to minimize potential bias and improve the rigor of statement selection, which allowed expert consensus to guide the finalization of statements [11,12]. Literature reviews and interviews yielded a diverse range of perspectives. The validation and refinement of the selected statements were performed by following the modified Delphi approach. The repetitive Delphi rounds facilitated expert feedback and revisions to ensure an accurate reflection of ICU nurses’ challenges and complexities in the final Q sample [10]. However, achieving consensus among experts with varying experience levels and perspectives on ICU workflows was the major challenge of the study.

The availability of the limited relevant literature is another main challenge. Though the nurse floating among ICU and non-ICU nurses has been a common phenomenon for decades, only a few recent studies have directly elaborated on it. Instead, the literature discusses it indirectly [6,8]. However, after COVID-19, the investigations have focused on assessing the non-ICU nurses’ perspectives regarding managing stress and caring for critically ill patients [4,7]. ICU nurses have often been asked about their experiences in supporting and mentoring non-ICU nurses, but it has never been the central focus of the studies [5]. Semi-structured interviews offered a broad range of perspectives. Direct interaction with ICU nurses about their experiences regarding working with non-ICU nurses revealed various challenges, which require further investigations for a deeper understanding [3,17]. During interviews, ICU nurses expressed communication barriers, differential skills, and additional responsibility of non-ICU nurses’ mentoring while simultaneously managing critically ill patients. Many ICU nurses revealed learning eagerness in non-ICU nurses; however, it often requires significant supervision that exerts extra stress and workload on ICU staff.

Researcher bias might lead to the type of statement selection that only reflects their expectations, and it could potentially yield biased results [9,10,18]. Therefore, a structured three-step approach was adopted during this study to construct the Q sample. The final statements’ selection was performed with the Delphi technique, including a panel of nursing experts of different levels who were familiar with ICU duties and had worked with non-ICU nurses. This technique helped reduce researcher bias and ensured collective decision-making by experienced ICU nursing workflow experts. Thus, it represented various nursing levels from supervisors to collaborative workers with non-ICU nurses. The Delphi panel participated in content validation, representativeness, and Q sample clarity. Moreover, experts provided feedback and suggested statements to improve the rigor of the selection process.

The low retention rate among expert panels could impact the Delphi technique’s rigor as it directly influences reaching a consensus. Participants’ unfamiliarity with the Delphi method could be responsible for this limitation, as some panelists might not have understood the necessity of multiple rounds [18,19]. Therefore, the research team sent written instructions via WhatsApp messages and email in addition to providing personal verbal explanations over the phone. These practices were performed to improve experts’ participation [20]. However, innovative approaches such as visual aids and the Delphi method explanation, through short videos, could further enhance experts’ understanding and retention in future studies. The extended period (two months) between Delphi rounds also contributed to a low retention rate. Prolonged intervals are known to alleviate participants’ engagement as they might perceive it as time-consuming and lose interest [14,20,21]. Therefore, a rapid round succession with clearly communicated timelines could facilitate participants’ better engagement and improved response rates.

## 5. Conclusions and Limitations

In conclusion, the development of an unbiased and comprehensive Q sample required a systematic and reflective approach. This study adopted a structured methodology combining literature reviews, semi-structured interviews, and the modified Delphi technique to ensure the inclusion of diverse and relevant perspectives. Despite the challenges of the limited literature and difficulty achieving expert consensus, the iterative Delphi process allowed for expert-driven refinement of the statements. The direct input from ICU nurses through interviews further enriched the Q sample by uncovering real-world experiences and challenges in working with non-ICU nurses. Researcher bias and expert panel retention were key concerns, but careful planning, clear communication, and follow-up strategies helped to mitigate these issues. Future studies should consider using more engaging methods, such as visual aids or short explanatory videos, to improve understanding and participation in Delphi rounds. A more compact timeline between rounds could also support better retention and response rates. Overall, the process adopted in this study contributed to the development of a credible Q sample that reflects the complexities of ICU nurses’ experiences when collaborating with non-ICU nurses in critical care environments.

Several limitations should be considered when interpreting the findings of this study. One of the primary limitations was the availability of the relevant literature directly addressing the dynamics between ICU and non-ICU nurses. Although floating nurses have been a common practice for decades, most existing studies have only addressed this issue indirectly, making it challenging to extract focused insights relevant to the current research objectives.

Another significant limitation was the difficulty in achieving and maintaining expert panel participation during the Delphi rounds. The low retention rate among panelists, possibly influenced by unfamiliarity with the Delphi method and the extended time intervals between rounds, affected the consistency of feedback and delayed consensus. Although multiple efforts were made to enhance participation, such as verbal explanations, WhatsApp messages, and email instructions, some participants may have found the process time-consuming or unclear. These factors may have limited the breadth of expert input, particularly in later rounds.

Researcher bias also remains a potential limitation, especially during the initial stage of statement selection. Although a structured three-step process and Delphi validation were implemented to minimize this bias, the initial filtering of interview data and the literature may have still reflected some level of subjective influence.

Lastly, while the semi-structured interviews offered valuable insights, the study was limited by the number of participants and the diversity of ICU settings represented. Therefore, the findings may not fully capture the range of experiences across different healthcare systems or geographic regions.

## 6. Ethical Considerations

Ethical approval for this study was obtained from the Institutional Review Board (IRB) of King Saud University, Riyadh, KSA No.: KSU-HE-23-1241 on 12 December 2023. The study was conducted in accordance with the ethical principles outlined in the Declaration of Helsinki. Participation was entirely voluntary, and all participants were informed that they could withdraw from the study at any point without any consequences. There were no anticipated risks associated with participation. Informed consent was obtained from all participants prior to data collection.

To ensure confidentiality and minimize the potential influence of dominant personalities during the Delphi rounds, participant anonymity was strictly maintained. The identities of the participants were known only to the primary researcher and were not shared during the data analysis or reporting stages. All data were stored securely in password-protected digital files accessible only to the research team. Experts were initially contacted and invited via WhatsApp or phone call, and upon agreement, the survey link was distributed through either WhatsApp or email according to their preference.

## Figures and Tables

**Table 1:** Statements included in the study.

No.	Statements
1. Preparation and Training	
1.	The institutions must prepare non-ICU nurses before floating to the ICU.
2.	The non-ICU nurses must prepare themselves before floating to the ICU.
3.	Non-ICU nurses need emotional support more than ICU competencies preparation.
4.	Redemonstrating common ICU procedures to non-ICU nurses is overwhelming.
5.	Non-ICU nurses require more time to be familiar with basic ICU knowledge.
6.	Nurses need special training to work in the ICU.
7.	The non-ICU nurses should learn and improve by themselves.
2. Collaborations and Communication	
8.	ICU nurses are constantly emphasizing the difference in practice between the ICU and the general ward to non-ICU nurses.
9.	Dealing with non-ICU nurses is time-consuming.
10.	Effective communication between ICU and non-ICU nurses is challenging.
11.	Non-ICU nurses resist learning ICU protocols.
12.	ICU nurses have difficulties dealing with non-ICU nurses in the unit.
13.	There is a concern when ICU policies and procedures are applied by a non-ICU nurse in the ICU unit.
3. Workload, Roles, and Dynamics	
14.	While ICU nurses are caring for their own patients, they need to lead non-ICU nurses.
15.	The role of ICU nurses is just caring for their own patients.
16.	The ICU and non-ICU nurses have the same job duties.
17.	Workload distribution should be equal between ICU and non-ICU nurses.
18.	ICU head nurses and unit managers promote ICU staff to work with non-ICU nurses.
19.	ICU nurses consider non-ICU nurses as beginners in ICUs.
20.	ICU nurses consider non-ICU nurses as an assistant to them in ICUs.
4. Feelings and Emotions	
21.	ICU nurses feel stressed when they are working with non-ICU nurses.
22.	ICU nurses are relaxed while they are working with non-ICU nurses.
23.	Working with non-ICU nurses makes ICU nurses exhausted.
24.	ICU nurses feel comfortable when they are working with non-ICU nurses.
25.	I deeply empathize with the non-ICU nurses for working in such a complex environment.
5. Patient Safety and Care Quality	
26.	Non-ICU nurses cannot detect minor changes that could affect a patient’s condition.
27.	Non-ICU nurses are not able to predict patient outcomes following specific interventions.
28.	It is safe for non-ICU nurses to handle unstable patients.
29.	Patient outcomes will be affected when handled by non-ICU nurses.
30.	Medical errors could increase when non-ICU nurses work in ICU units.
31.	Lack of cooperation from non-ICU nurses could compromise patient safety.
32.	Working with non-ICU nurses interferes with the quality of patients’ care.
6. Opportunities and Trust	
33.	ICU nurses learned a lot from dealing with non-ICU nurses.
34.	ICU nurses are trusted more than non-ICU nurses.
35.	It is easy to deal with non-ICU nurses who are interested in ICU units.
36.	Non-ICU nurses are familiar with ICU protocols.
37.	ICU nurses should share their knowledge and experience with non-ICU nurses.
38.	Non-ICU nurses, with their diverse experience, could add value to ICU situations.
39.	ICU nurses believe in non-ICU nurses’ efforts for the benefit of patients.
40.	It is a great opportunity to mentor non-ICU nurses who have experience in their own units.

Source: Elaborated by the authors.

## Data Availability

All data generated or analyzed during this study are included in this published article. No additional data are available.

## Supplementary Materials

The following supporting information can be downloaded at: https://www.mdpi.com/article/10.3390/healthcare13090973/s1, Supplementary Material: Sample of the coded data and extracted statements.
