# Supplementary material for: Developing a Q Set Using the Modified Delphi Technique to Investigate ICU Nurses’ Perspectives on Working with Non-ICU Nurses
Source: Healthcare (Basel). 2025 Apr 23;13(9):973. doi: 10.3390/healthcare13090973 (PMC12071961; doi:10.3390/healthcare13090973)
Supplement: Supplementary file 1 [file healthcare-13-00973-s001.zip › healthcare-3553533-supplementary.pdf]

**Supplementary Materials**

Sample of the coded data and extracted statements

| Participant Quote | Initial code | Statement extracted |
|---|---|---|
| (P9) I had to take on some teaching responsibilities. I must say, the ward staff were under a lot of mental stress — they were really afraid of dealing with ICU patients | 1-Teaching responsibility<br>2- Non-ICU mental stress | ICU nurses are responsible for sharing their knowledge and experience with inexperienced nurses<br><br>I empathize deeply with the non-ICU nurses working in such a complex environment. |
| (P5) We assign them a patient and then explain our ICU routines, including doctors' rounds. We also go over what to do if a surgical or trauma patient arrives — we need to explain everything to them. | Explain everything | Non-ICU nurses require plenty of preparation before working with critically ill patients. |
| (P1) we got many unit ward staff to ICU. It was really challenging time for us | Challenges | It is challenging to deal with non-ICU nurses |
| (P1) I was think about my initial days when I will go for float from ICU to ward. Really it was hard. There is stress in my mind how to deal instead of one patient six patients even though they are stable six patients. How it was. So it just came in my mind that it is how the almost the same feeling may also be for them | Feeling their feelings | I empathize deeply with the non-ICU nurses working in such a strange environment |
| (P4) I felt really happy — like they (non-ICU nurse) were accepting the situation. During the COVID pandemic, there was a staff shortage, and they had to take it on as their own responsibility. That gave me a boost of energy. I'm really telling you, it was a real boost. | Happy and excitement | ICU nurses are excited and happy when working with non-ICU nurses |
| (P9) With limited resources and limited (ICU) manpower, (ICU nurses) they were really under a lot of stress | Under stress | It is stressful working with non-ICU nurses when there is difficult situations |
| (P4) They (ICU nurses) need to care for very critical patients, while at the same time, non-ICU staff are handling less critical | Monitoring and supervision | ICU nurses need to supervise non-ICU nurses while handling their own patient |

| | | |
|---|---|---|
| ones. So, the non-ICU staff need to be closely monitored for a period of time." | | |
| (P4) They (ICU nurses) need to care for very critical patients, while at the same time, non-ICU staff are handling less critical ones. So, the non-ICU staff need to be closely monitored for a period of time." | Workload acuity | It is unfair for ICU nurses are handling more critically patient while non-ICU handle stable patient |
| (P6) They are pulled out to ICU, there is some kind of resistance and they want to go back to their own units. Such feeling was there | Resistance | it is difficult to work with Non-ICU nurses as they resist working in the ICU |
| (P5) My patient went into cardiac arrest and no one was with me. One colleague was nearby, but she didn't help. I started chest compressions alone, and then I called for help. She didn't respond at that time — maybe it was her first time in that situation, I don't know — but she didn't help. | Lack of cooperation | Non-ICU nurses are careless when it comes to teamwork |
| (P6) It was the first time that I felt very angry. ... I cannot do three patients. Only two is okay. This is maximum care... But of course, ICU more than the non ICU stuff. | Workload distribution number | It is unfair to handle more critically ill patient than the no-ICU |
